# News and Notes

**Published:** 1904-07

**Authors:** 


### NEWS AND NOTES.

BUBONIC PLAGUE continues on the west coast of South America.

---

MILAN, Italy, will soon establish a "Bassini Institute" to supply operation treatment of hernia to the poor.

---

PLAGUE has broken out among the Chinese collected at Kowloon for transportation to the Transvaal mines.

---

THE twenty-ninth annual meeting of the American Gynecological Society was held in the Medical Library, Boston, on May 24, 25 and 26.

---

MISS CLARA BARTON has resigned the presidency of the Red Cross Society. A new charter will be issued, with various changes in the plan of management.

---

ALL the Esquimaux living in the Mackenzie basin except ten families have been killed by the ravages of measles. Before the epidemic there were forty or fifty families, or two hundred or more persons.

---

DR. L. AMSTER is in Europe taking courses in gastric and gastro-intestinal diseases. Before returning to Atlanta he expects to spend some time with Einhorn in New York, and Hemmeter in Baltimore.

DR. WALTER LINDLEY, the editor of the *Southern California Practitioner*, has recently been elected Dean of the Medical College of the University of Southern California. This Los Angeles school is now entering its twentieth session. Dr. Lindley was one of the organizers of the school and is Professor of Gynecology in that institution.

---

IN Zurich a lady doctor has been advocating that all unmarried women of the well-to-do classes should be compelled by the State to devote one year to unpaid hospital work. She claims that the hospitals would benefit and the girls gain a training of great value to them after marriage. The latter may be true, but the results to the hospitals would, we imagine, be anything but desirable.

---

AN exchange says: "Habitual drunkards after conviction in Montreal, Quebec, are now given the option of paying a fine, undergoing imprisonment, or taking a certain cure. At present there are twenty-two under treatment, ten at their homes and twelve at the jail. Each patient is expected to take sixteen doses of the prescribed medicine each day, and is warned not to drink any intoxicating liquors during the time of trial."

---

THE Chinese national intoxicant is named "Chansin." It is obtained principally from an herb called Gaolyan, which is grown on almost every peasant's land in Manchuria, being used partly for cattle food. Chansin is not uniform in spirit contents, but may be taken to be about 150 per cent. stronger than Russian vodka. It is prepared in very old-fashioned apparatus. Poisonous impurities are often present in appreciable quantities.

---

DR GERSUNY, who now is perhaps the best known of all Billroth's pupils, recently celebrated his sixtieth birthday in a fashion different to that common in Austria. Instead of holding a reception he performed four prolonged operations. It is to Gersuny that in Austria is ascribed the credit of several new operations and modifications of technique, especially in connection with plastic operations on the vagina, the use of paraffin injections, and the introduction of torsion to produce an artificial sphincter ani.

---

MR. CARNEGIE has created a fund of $5,000,000 for the benefit of persons dependent on those who have lost their lives in heroic efforts to save life, or of such persons themselves if injured but not killed. The benefits of the fund apply to Canada and the United States, and Mr. Carnegie's letter contains the following: "No action (is) more heroic than that of doctors and nurses volunteering their services in the case of epidemics. . . . All these and similar cases are embraced."

---

THE following cable despatch recently appeared in the New York *Sun*: "Ten years ago the injection of salt water as a restorative to patients dying from loss of blood aroused general interest. The discovery of the quality of salt water probably suggested to the French savant, M. Quinton, a long and patient research concerning salt water, the conclusion of which throws unexpected light on and adds support to the Darwinian theory of evolution. M. Quinton maintains that sea water is the natural source from which, as Professor Haeckel believes, elementary bodies rise which develop into all the species, including the human. The environment wherein the anatomical elements of living creatures exist is neither more nor less than a marine one. Our tissues and cells continue to exert their functions in a fluid, the composition of which bears the closest resemblance to that of sea water."

